# Rate-Dependent Tensile Properties of Aluminum-Hydroxide-Enhanced Ethylene Propylene Diene Monomer Coatings for Solid Rocket Motors

**DOI:** 10.3390/ma17153790

**Published:** 2024-08-01

**Authors:** Ran Wang, Yiming Zhang, Ningfei Wang, Yi Wu

**Affiliations:** 1 School of Aerospace Engineering, Beijing Institute of Technology, Beijing 100081, China; wr.wangrran@bit.edu.cn (R.W.);; 2 Chongqing Innovation Center, Beijing Institute of Technology, Chongqing 401120, China

**Keywords:** solid rocket motor, aluminum-hydroxide-enhanced EPDM, tensile constitutive model, particle swarm optimization

## Abstract

Quasi-static and dynamic tensile tests on aluminum-hydroxide-enhanced ethylene propylene diene monomer (EPDM) coatings were conducted using a universal testing machine and a Split Hopkinson Tension Bar (SHTB) over a strain rate range of 10^−3^ to 10^3^ s^−1^. This comprehensive study explored the tensile performance of enhanced EPDM coatings in solid rocket motors. The results demonstrated a significant impact of strain rate on the mechanical properties of EPDM coatings. To capture the hyperelastic and viscoelastic characteristics of EPDM coatings at large strains, the Ogden hyperelastic model was used to replace the standard elastic component to develop an enhanced Zhu–Wang–Tang (ZWT) nonlinear viscoelastic constitutive model. The model parameters were fitted using a particle swarm optimization (PSO) algorithm. The improved constitutive model’s predictions closely matched the experimental data, accurately capturing stress–strain responses and inflection points. It effectively predicts the tensile behavior of aluminum-hydroxide-enhanced EPDM coatings within a 20% strain range and a wide strain rate range.

## 1. Introduction

Ethylene propylene diene monomer (EPDM) composite is a type of carbonized ablative material made by incorporating fillers into an EPDM matrix, mixing with other additives, and vulcanizing [1]. Due to its excellent aging resistance, corrosion resistance, and thermal insulation properties, EPDM composite has been widely used in aerospace engineering [2,3,4,5,6,7]. Specifically, in solid rocket motors, this material is typically used as an insulating coating to protect the mechanical properties of the engine casing from the high temperatures of the combustion chamber, which is positioned between the case and the propellant grain [8,9]. With increasing attention being paid to the integrity of solid rocket motor propellant structures in recent years, the importance of the structural integrity of the coating, as a critical part of the propellant structure, has become more pronounced [10,11,12,13,14,15]. Therefore, studying the mechanical behavior of EPDM composite coating materials and developing accurate constitutive models for their mechanical properties will provide a theoretical basis for addressing the structural integrity issues of solid rocket motors using EPDM composite as the coating.

In recent years, many researchers have conducted studies on the thermal properties of EPDM composite from the perspective of material composition, investigating the effects of various additives. George et al. [16] investigated the effect of aramid fiber addition on surface morphology and density, analyzing parameters such as thermal conductivity, heat capacity, and thermal diffusivity. Li et al. [17] studied the significant impact of silica fillers and aramid fibers on the ablation resistance of EPDM composites, finding that the combination of silica and aramid fibers enhanced the material’s thermal insulation and erosion resistance. Igor et al. [18] examined the influence of reinforcing fiber orientation on the ablation and erosion performance of EPDM insulation materials, discovering that longitudinal fiber orientation exhibited the least ablation resistance. Additionally, Jia [19,20], Gao [21], Vishvanathperumal [22], and Arshad [23] have found that high-performance fibers, such as polysulfonamide (PSA) and polyimide (PI), exhibit greater ablation resistance. Natali [24] evaluated the performance of wollastonite as a harmless mineral substitute for aramid fibers in EPDM composites, revealing that wollastonite-reinforced EPDM materials exhibited slightly lower insulation capability and some density loss. Zhang et al. [25] investigated the effects of silicon carbide whisker (SiCw) and montmorillonite (MMT) fillers on enhancing the ablation resistance of composites.

In terms of mechanical properties, Fan et al. [26] characterized the quasi-static deformation behavior of EPDM composites through uniaxial tensile tests and stress relaxation tests, proposing a hyper-viscoelastic constitutive model with energy limiters. Jiang et al. [27] studied the rate-dependent compressive mechanical behavior of EPDM insulation materials and developed an improved nonlinear visco-hyperelastic compressive constitutive model. Wang et al. [28] measured the mechanical properties of thermally aged EPDM coatings through uniaxial tensile and hardness tests, obtaining stress–strain curves and mechanical parameters of EPDM coatings under different aging temperatures and times, and established an aging hyperelastic constitutive model for EPDM coatings. Song et al. [29] evaluated the mechanical behavior of EPDM under dynamic compression and modified the one-dimensional dynamic constitutive equation based on strain energy function theory. Cheng et al. [30] studied the tensile behavior of EPDM matrices, finding a Mullins effect in the stress–tensile behavior under dynamic loading. Enew et al. [31] investigated the effects of aramid and nanocarbon particle fibers on the mechanical properties of EPDM insulation materials for solid rocket motors. Li et al. [32] found that nanocrystalline aerogel (rGCA)/EPDM composites (rGCA/EPDM) exhibit excellent compressibility and recoverability. Xu et al. [33] discovered that the addition of TA@SiNf/PGNS significantly enhanced the material’s mechanical strength and fracture toughness.

In summary, various fillers significantly impact the ablation resistance and mechanical properties of EPDM composites. Specifically, for aluminum-hydroxide-enhanced EPDM composites used in solid rocket motors, the high aluminum hydroxide content contributes to excellent flame retardancy. However, the poor adhesion between aluminum hydroxide particles and the matrix can negatively affect the tensile properties of the composites [34,35,36]. Currently, there is no systematic research on the tensile properties of aluminum-hydroxide-enhanced EPDM coating materials for solid rocket motors. During practical use, solid rocket motors undergo production, transportation, storage, and launch, experiencing both quasi-static and dynamic loads. Therefore, it is crucial to study the tensile mechanical properties of enhanced EPDM coatings across a wide range of strain rates and develop an appropriate constitutive model.

In this study, both quasi-static and dynamic tensile experiments were conducted on aluminum-hydroxide-enhanced EPDM coating composites using a universal testing machine and a Split Hopkinson Tension Bar (SHTB) to comprehensively investigate the tensile properties of EPDM coatings in solid rocket motors. The Ogden hyperelastic model was used to replace the standard elastic component in developing an enhanced ZWT nonlinear viscoelastic constitutive model, with model parameters fitted using a particle swarm optimization algorithm. The improved constitutive model effectively predicted the tensile mechanical behavior of the aluminum-hydroxide-enhanced EPDM coating materials, facilitating the development of reliable numerical methods to address structural issues in solid rocket motors using aluminum-hydroxide-enhanced EPDM as an insulating material.

## 2. Materials and Methods

### 2.1. Materials and Specimens

The aluminum-hydroxide-enhanced ethylene propylene diene monomer (EPDM) coating composite material used in this study, with EPDM rubber as the base, exhibited excellent ablation resistance and erosion resistance. As detailed in Table 1, it comprised 56 wt% EPDM, 30 wt% aluminum hydroxide (Al(OH)_3_), 10 wt% liquid flame retardant, and 4 wt% processing aids, all sourced from the Xi’an Modern Chemistry Research Institute (Xi’an, China).

**Figure 1:**
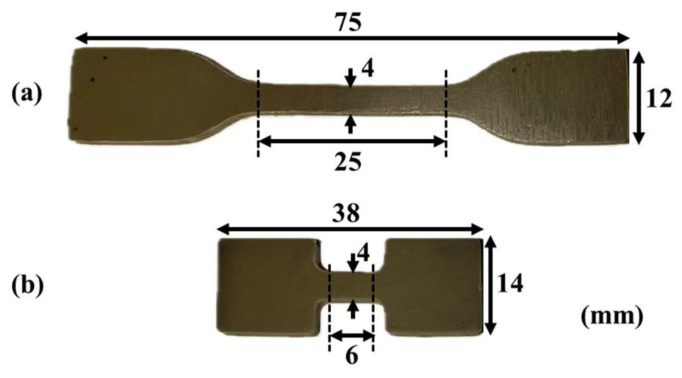
Specimen specifications: (**a**) quasi-static tensile specimen; (**b**) specimen under 1000–3000 s^−1^ dynamic tensile strain rate.

**Figure 2:**
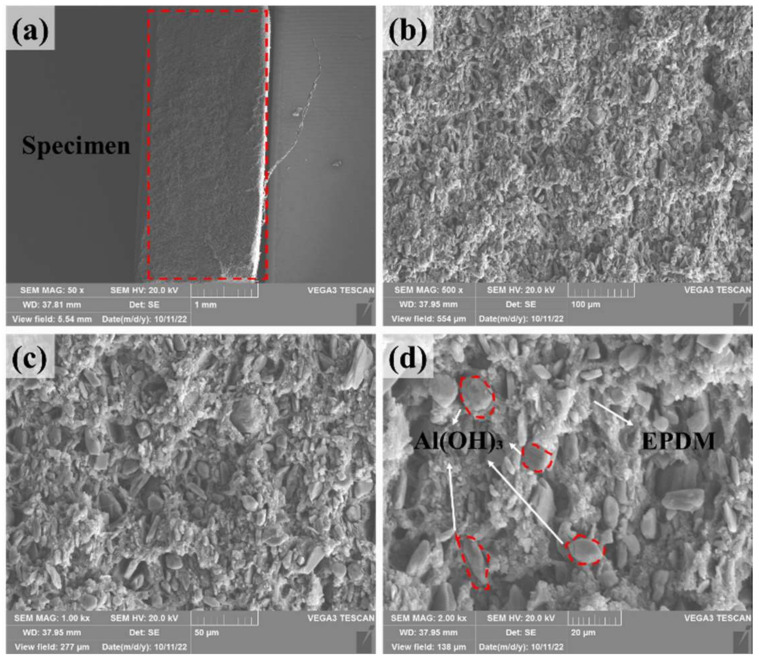
SEM images of EPDM coating specimen: (**a**) 50×; (**b**) 500×; (**c**) 1000×; (**d**) 2000×. The red dashed box in (**a**) indicates the specimen mentioned in the text. The red circles in (**d**), as indicated by the arrow, represent Al(OH)_3_.

As shown in Figure 1, the specimens were prepared using two different molds to accommodate both quasi-static and dynamic loading strain rates, with a thickness of 2 mm. Figure 2 presents the scanning electron microscope (SEM) images of the EPDM coating specimens’ cross-sections at various magnifications, revealing the uniform distribution of aluminum hydroxide particles within the matrix. After preparation, the specimens were stored under constant temperature and humidity conditions for five days prior to testing to alleviate any residual stresses introduced during processing.

### 2.2. Quasi-Static Experiments

**Figure 3:**
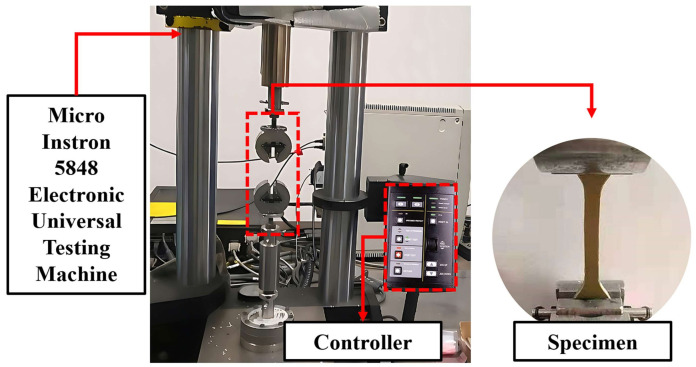
Schematic diagram of universal tensile testing machine.

As shown in Figure 3, the quasi-static tensile mechanical performance tests were conducted at room temperature (293 K) using a Micro Instron 5848 universal testing machine sourced from Instron, Norwood, MA, USA. Different tensile strain rates were achieved by controlling the crosshead speed of the Micro Instron 5848. During the experiments, a video extensometer was utilized to measure strain to ensure the validity of the experimental results. At least three specimens were tested for each strain rate condition to ensure good repeatability.

According to the principle of constant volume, the true values of engineering strain rate, engineering strain, and engineering stress under tension can be calculated using Equation (1).

(1) $$\left\{\begin{array}{l} \varepsilon =\varepsilon_{t}=\ln\left(1+\varepsilon_{e}\right)\\ \sigma =\sigma_{t}=\sigma_{e}\left(1+\varepsilon_{e}\right)\\ \dot{\varepsilon }={\dot{\varepsilon }}_{t}={\dot{\varepsilon }}_{e}/\left(1+\varepsilon_{e}\right) \end{array}\right.$$

where ${\dot{\varepsilon }}_{t}$ is the true strain rate, ${\dot{\varepsilon }}_{e}$ is the engineering strain rate, $\varepsilon_{t}$ and $\varepsilon_{e}$ are the true strain and engineering strain, and $\sigma_{t}$ and $\sigma_{e}$ represent the true stress and engineering stress.

### 2.3. Dynamic Experiments

**Figure 4:**
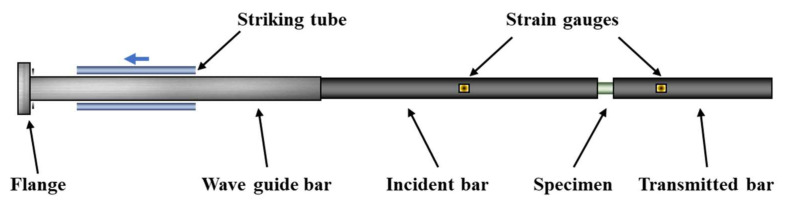
Schematic diagram of SHTB.

The dynamic tensile tests were conducted at room temperature (293 K) using a modified Split Hopkinson Tensile Bar (SHTB) setup, as schematically illustrated in Figure 4. The apparatus consisted of a gas cylinder, striking tube, flange, waveguide bar, incident bar, transmitted bar, and a data acquisition system. The specimen was affixed in a pre-prepared groove between the incident and transmitted bars. Unlike the traditional Hopkinson Tension Bar, the transmitted bar in this study was made of polymethyl methacrylate (PMMA) instead of the conventional metal material used for the incident bar. The choice of PMMA, which has a lower elastic modulus than metal, facilitates the detection of weak signals from low-impedance specimens [37]. During the tests, it is essential to ensure that the striking tube, incident bar, transmitted bar, and specimen are aligned coaxially and horizontally. Additionally, strain gauges are radially attached at the midpoint of the incident bar and near the front end of the transmitted bar close to the specimen to measure the strain signals throughout the experiment.

During the experiment, a certain amount of nitrogen gas was first filled into the cylinder. Once the gas pressure stabilized, the launching device was quickly activated. The nitrogen gas in the cylinder propelled the striking tube to hit the flange at a certain speed, generating a compressive stress wave in the flange. This stress wave reflected as a tensile wave at the free end of the flange and propagates through the waveguide bar to the incident bar. The amplitude of the stress wave was proportional to the impact speed. When the incident wave reached the specimen, due to impedance mismatch, part of the stress wave was reflected back along the incident bar as a compressive wave, while the other part transmitted through the specimen into the transmitted bar. Different strain rates were achieved by adjusting the gas pressure in the gas gun and using striking tube of different lengths.

Strain gauges were connected to a bridge box (using the Wheatstone half-bridge principle) to convert the strain signals generated by the stress waves in the bars into voltage signals. These voltage signals were then recorded by a data acquisition system. The strain gauges on the incident bar collected incident and reflected signals, while those on the transmitted bar collected transmitted signals. It is important to note that the transmitted signal measured by the metal strain gauges was very weak and noisy, so semiconductor strain gauges with a higher gain factor were used to collect the transmitted signal in this experiment [38,39].

The parameters of each component in the Hopkinson bar experimental setup are shown in Table 2. The striking tube was a hollow rod with an outer diameter of 27 mm and an inner diameter of 20 mm. The resistance of the metal strain gauge on the incident bar was 1000 Ω with a sensitivity coefficient of 1.92, and it was connected to a 30 V DC power supply. The semiconductor strain gauge on the transmission bar had a resistance of 120 Ω with a sensitivity coefficient of 110, and it was connected to a 5 V DC power supply.

## 3. Results and Discussion

### 3.1. Data Validation of Dynamic Experiments

**Figure 5:**
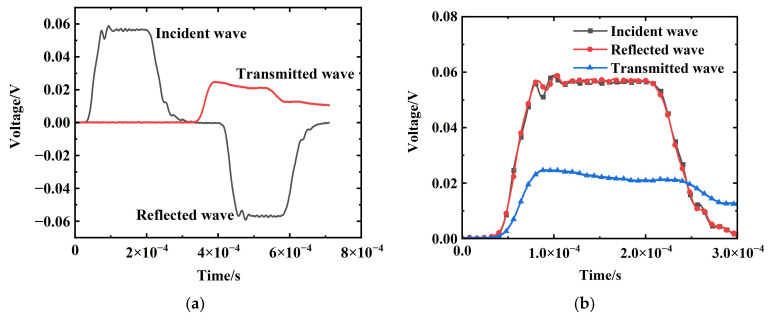
Typical set of incident, reflected, and transmitted signals: (**a**) raw signals; (**b**) wave-form separation figure.

Figure 5 presents a set of the typical incident, reflected, and transmitted signals during the SHTB experiment on an EPDM coating specimen at a strain rate of 2500 s^−1^. The high-frequency components resulting from the high-velocity impact on the incident bar were not evident in the incident signal. The plateau-like region observed in the reflected signal indicated the deformation of the sample under a constant engineering strain rate. In Figure 5a, the minute oscillations shown signify weak interference signals, which are eliminated through filtering, rendering their impact on the experimental results negligible. Due to the extremely weak signals obtained from the metallic strain gauges in the transmitted signal and the considerable noise, this experiment utilized signals obtained from the semiconductor strain gauges for the calculations. The conversion formula for voltage signal to strain signal is as follows:

(2) $$\varepsilon_{T}=\frac{250}{R\cdot K}\cdot \frac{\Delta U}{U-\Delta U}=\frac{250}{120\times 104}\cdot \frac{\Delta U}{5-\Delta U}$$

where *R* is the resistance of the strain gauge, *K* is the sensitivity coefficient of the strain gauge, and *U* is the voltage of the connected power supply.

According to one-dimensional stress wave theory, the engineering strain rate, engineering strain, and engineering stress can be calculated using Equation (3). The true values are also calculated using Equation (1) [40,41].

(3) $$\left\{\begin{array}{l} {\dot{\varepsilon }}_{e}=\frac{1}{L_{s}}\left(C_{I}\varepsilon_{I}-C_{I}\varepsilon_{R}-C_{T}\varepsilon_{T}\right)\\ \varepsilon_{e}=\int_{0}^{t}{\dot{\varepsilon }}_{e}d\tau =\frac{1}{L_{s}}\int_{0}^{t}\left(C_{I}\varepsilon_{I}-C_{I}\varepsilon_{R}-C_{T}\varepsilon_{T}\right)d\tau \\ \sigma_{e}=\frac{1}{2A_{s}}\left(A_{I}E_{I}\varepsilon_{I}+A_{I}E_{I}\varepsilon_{R}+A_{T}E_{T}\varepsilon_{T}\right) \end{array}\right.$$

where *C* represents the wave speed, *E* stands for the elastic modulus, and *L* and *A* denote length and cross sectional area, respectively. The subscripts *I*, *T*, and *S* correspond to the incident bar, transmitted bar, and specimen, respectively. $\varepsilon_{I}$, $\varepsilon_{R}$, and $\varepsilon_{T}$ represent the incident strain signal, reflected strain signal, and transmitted strain signal calculated using Equation (2), respectively.

**Figure 6:**
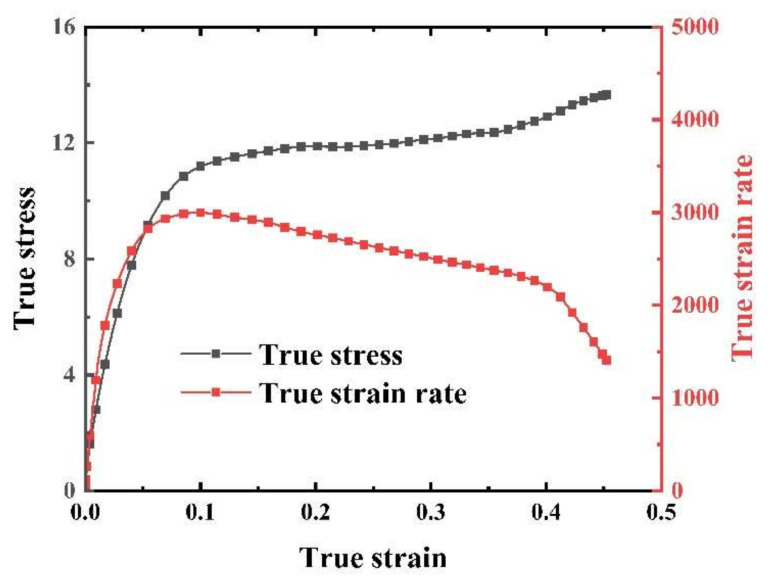
Stress–strain curves and strain rate–strain curves at 2500 s^−1^.

The strain rate history at 2500 s^−1^ is depicted in Figure 6. It is observable that the strain rate curve presents a plateau, indicating that the sample was primarily subjected to constant strain rate loading. Hence, it meets the requirements for nearly ideal transmitted waves and uniform strain rates, affirming the reliability of the experiment [42]. However, before reaching a deformation of 0.1, there was a rapid increase in strain rate, indicating inaccuracies in the experimental results during this period. Therefore, our focus was on the mechanical behavior of the EPDM specimen under constant strain rate conditions.

### 3.2. Stress–Strain Results

**Figure 7:**
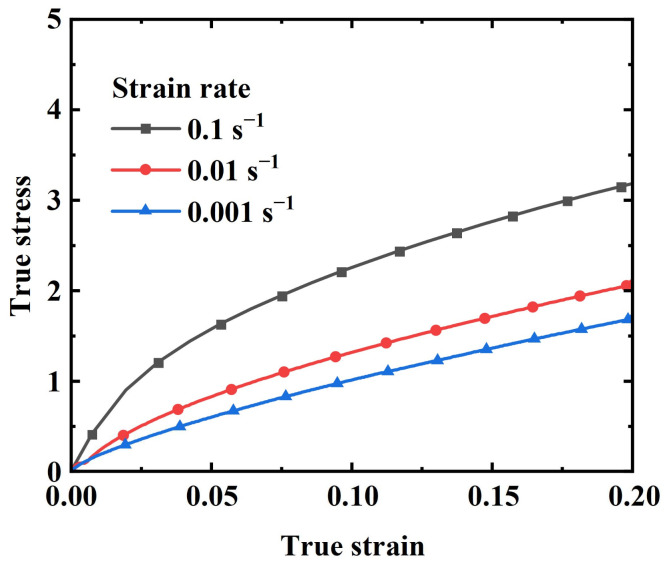
Quasi-static tensile stress–strain curves of EPDM coating specimen.

**Figure 8:**
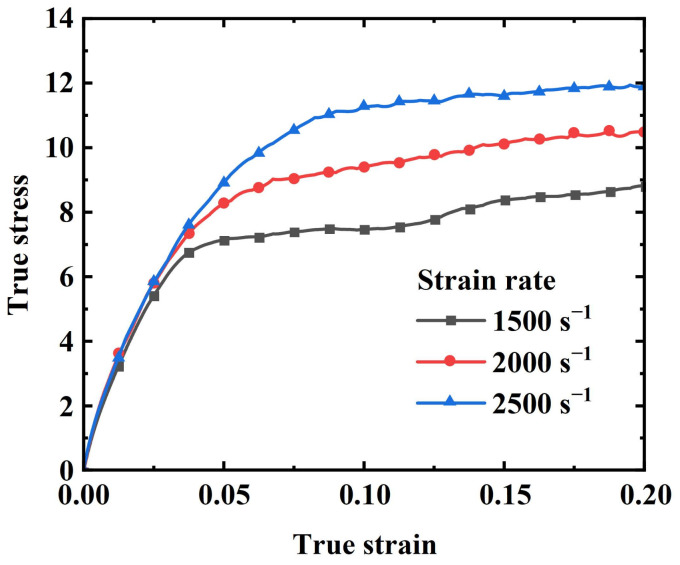
Dynamic tensile stress–strain curves of EPDM coating specimen.

As shown in Figure 7 and Figure 8, tests were conducted at three different strain rates for both quasi-static and dynamic conditions, presenting the true stress–true strain curves within a 20% strain range for each strain rate. As can be seen from the figures, there are some differences between the quasi-static and dynamic test curves. At lower strain rates (e.g., 0.001 s^−1^), the stress–strain curve exhibits a strong linear relationship. As the strain rate increases, a distinct inflection point begins to appear in the curve (Figure 7). The inflection point phenomenon is even more pronounced in the dynamic tests (Figure 8), where the curves generally consist of two stages: a linear increase stage and a slow growth stage. Initially, the stress escalates swiftly in a linear manner, then gradually rises beyond the inflection point as the strain continues to increase. Both the strain and strain rate contribute to the rise in stress, with the stress value at the inflection point also increasing alongside the strain rate.

## 4. Tensile Constitutive Model

The Zhu–Wang–Tang (ZWT) nonlinear constitutive model has been widely applied in fields such as plastics and propellants, demonstrating its capability to characterize the mechanical properties of materials over a broad range of strain rates [43,44,45,46]. However, it has certain limitations, as the literature indicates that this model is reliable only for strains less than 8% [47]. Considering the hyperelasticity of the aluminum-hydroxide-enhanced EPDM coating, this study aimed to characterize the mechanical properties of the EPDM coating at larger strains by replacing the nonlinear elastic equilibrium response in the ZWT constitutive model with a hyperelastic response. This modification allowed for a more comprehensive description of the hyperelastic and viscoelastic properties of the coating material over a wide range of strain rates.

### 4.1. Nonlinear Viscoelastic Constitutive Model

The ZWT constitutive model consists of a nonlinear elastic equilibrium response and two Maxwell viscoelastic responses connected in parallel. The first Maxwell unit describes the quasi-static, low-strain-rate viscoelastic response, while the second Maxwell unit characterizes the dynamic, high-strain-rate viscoelastic response properties. The stress model in relation to the strain *ε* and its rate of change $\dot{\varepsilon }$ is represented as follows:

(4) $$\sigma =E_{0}\varepsilon +\alpha \varepsilon^{2}+\beta \varepsilon^{3}+E_{1}\int_{0}^{t}\dot{\varepsilon }(\tau )exp(-\frac{t-\tau }{\theta_{1}})d\tau +E_{2}\int_{0}^{t}\dot{\varepsilon }(\tau )exp(-\frac{t-\tau }{\theta_{2}})d\tau$$

where $E_{0}$, *α*, and *β* are the elastic constants; $E_{1}$ and $\theta_{1}$ represent the elastic constants and relaxation times for the first Maxwell unit, capturing the viscoelastic response at low strain rates. Meanwhile, $E_{2}$ and $\theta_{2}$ denote the corresponding elastic constants and relaxation times for the second Maxwell unit, illustrating the viscoelastic response at high strain rates. The variable *t* signifies the relaxation time.

### 4.2. Hyper-Viscoelastic Constitutive Model

Many hyperelastic constitutive models have been established both domestically and internationally to describe the mechanical behavior of elastic materials, such as the Neo–Hookean model, the Mooney–Rivlin model, the Yeoh model, and the Ogden model [48,49,50,51,52]. Among these, the Ogden model has been widely used in the computation of rubber or similar hyperelastic structures due to its high accuracy in simulating large deformation problems [53,54].

The Ogden model, proposed by Ogden, overcomes the complexity of relationships caused by using invariants of the deformation tensor by representing the strain energy function in terms of principal stretches. The form of the strain energy function is as follows:

(5) $$W=\sum_{n=1}^{\infty }\frac{2\mu_{n}}{\alpha_{n}^{2}}(\lambda_{1}^{\alpha_{n}}+\lambda_{2}^{\alpha_{n}}+\lambda_{3}^{\alpha_{n}}-3)$$

where $\mu_{n}$ and $\alpha_{n}$ are arbitrary constants determined through fitting using the particle swarm algorithm (PSO) [55,56].

The terms within the Ogden model can be adjusted based on experimental data to achieve a better fitting effect, thus providing greater flexibility. The expression for nominal stress–nominal strain in the Ogden model is as follows:

(6) $$\sigma_{e}=\sum_{n=1}^{\infty }\frac{2\mu_{n}}{\alpha_{n}}\left[{\left(1+\varepsilon_{e}\right)}^{\alpha_{n}-1}-{\left(1+\varepsilon_{e}\right)}^{-\alpha_{n}/2-1}\right]$$

Here, the correlation between true stress and true strain is established in line with Equation (1), and the expression is given here:

(7) $$\sigma_{e}=\sum_{n=1}^{\infty }\frac{2\mu_{n}}{\alpha_{n}}\left[e^{\varepsilon \alpha_{n}}-e^{-\varepsilon \alpha_{n}/2}\right]$$

### 4.3. Constitutive Model of EPDM Coating

In order to characterize the hyperelasticity and viscoelasticity of the aluminum-hydroxide-enhanced EPDM coating, the elastic equilibrium response unit within the ZWT constitutive model was replaced with the Ogden hyperelastic model, resulting in the development of the following new constitutive model:

(8) $$\sigma =\sum_{n=1}^{\infty }\frac{2\mu_{n}}{\alpha_{n}}\left[e^{\varepsilon \alpha_{n}}-e^{-\varepsilon \alpha_{n}/2}\right]+E_{1}\int_{0}^{t}\dot{\varepsilon }(\tau )exp(-\frac{t-\tau }{\theta_{1}})d\tau +E_{2}\int_{0}^{t}\dot{\varepsilon }(\tau )exp(-\frac{t-\tau }{\theta_{2}})d\tau$$

Under quasi-static compression conditions, the high-strain-rate Maxwell unit relaxes at the onset of loading, allowing the high-strain-rate integral term to be neglected, simplifying the equation to the following:

(9) $$\sigma =\sum_{n=1}^{\infty }\frac{2\mu_{n}}{\alpha_{n}}\left[e^{\varepsilon \alpha_{n}}-e^{-\varepsilon \alpha_{n}/2}\right]+E_{1}\int_{0}^{t}\dot{\varepsilon }(\tau )exp(-\frac{t-\tau }{\theta_{1}})d\tau$$

Conversely, during dynamic compression, the impact test duration is short, and the low-frequency Maxwell unit does not have sufficient time to relax until the loading ceases. Consequently, the Maxwell unit simplifies to a linear spring unit with a constant $E_{1}$. Thus, the mechanical behavior of EPDM coating under high-strain-rate loading simplifies to the following:

(10) $$\sigma =\sum_{n=1}^{\infty }\frac{2\mu_{n}}{\alpha_{n}}\left[e^{\varepsilon \alpha_{n}}-e^{-\varepsilon \alpha_{n}/2}\right]+E_{1}\varepsilon +E_{2}\int_{0}^{t}\dot{\varepsilon }(\tau )exp(-\frac{t-\tau }{\theta_{2}})d\tau$$

## 5. Model Construction and Verification

Utilizing the particle swarm algorithm, the parameters of the enhanced ZWT model were fitted to both static and dynamic compression experimental results, further investigating the tensile mechanical behavior of the EPDM coating. The findings indicate that the model can effectively predict the tensile mechanical properties of EPDM coating, even for strain rates not explicitly considered during the fitting process.

### 5.1. Parameter Calibration

**Figure 9:**
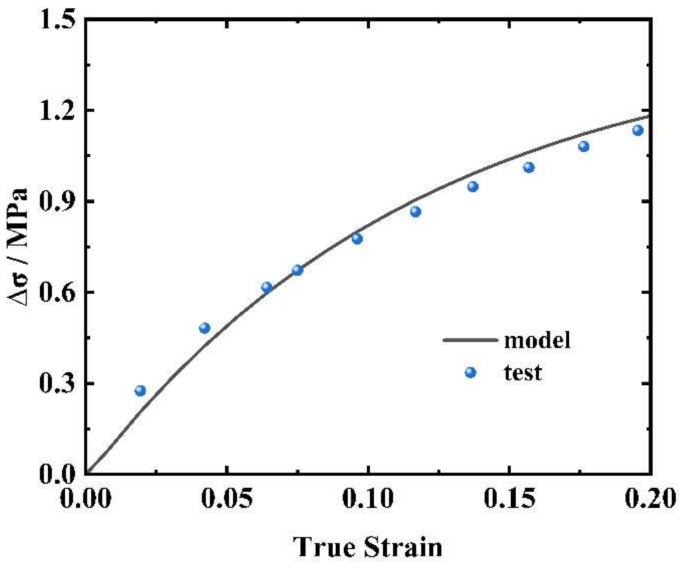
Fitting result for the difference between the static curves ∆σ.

The nonlinear viscoelastic constitutive model established in this study encompasses ten model parameters, comprising six hyperelastic parameters, two low-strain-rate response parameters ($E_{1}$ and $\theta_{1}$), and two high-strain-rate response parameters ($E_{2}$ and $\theta_{2}$). The differential curve was obtained by subtracting the experimental curves obtained at strain rates of 0.001 s^−1^ and 0.1 s^−1^. $E_{1}$ and $\theta_{1}$ were be obtained by fitting the difference to Equation (11). The results are depicted in Figure 9.

(11) $$\Delta \sigma =E_{1}\theta_{1}{\dot{\varepsilon }}_{1}(1-exp(-\frac{\varepsilon }{{\dot{\varepsilon }}_{1}\theta_{1}}))-E_{1}\theta_{1}{\dot{\varepsilon }}_{2}(1-exp(-\frac{\varepsilon }{{\dot{\varepsilon }}_{2}\theta_{1}}))2$$

**Figure 10:**
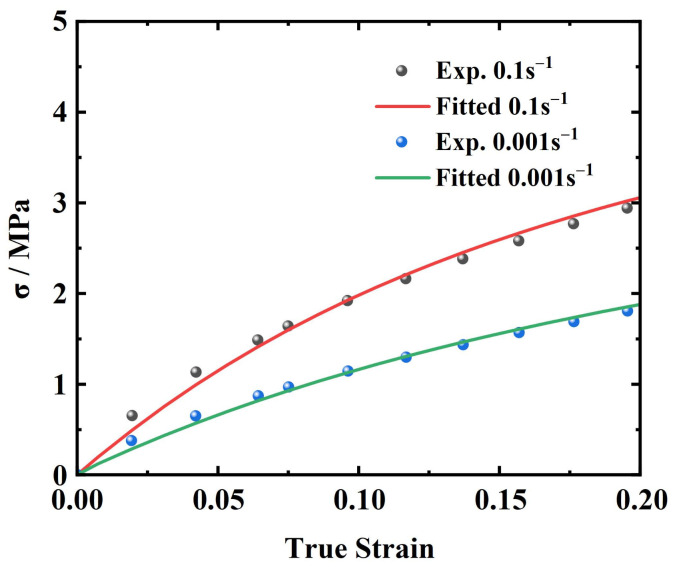
Fitting result for 0.001 s^−1^ and 0.1 s^−1^.

$\alpha_{1}$, $\mu_{1}$, $\alpha_{2}$, $\mu_{2}$, $\alpha_{3}$, and $\mu_{3}$ were obtained by fitting the quasi-static test data at a strain rate of 0.001 s^−1^ to Equation (9). The results are illustrated in Figure 10.

**Figure 11:**
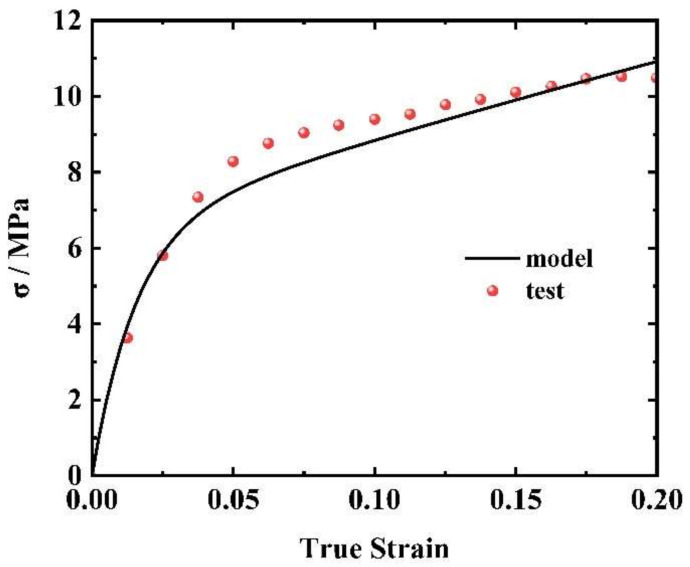
Fitting result for 2000 s^−1^.

The high-strain-rate response parameters $E_{2}$ and $\theta_{2}$ were obtained by fitting the stress–strain curve at a strain rate of 2000 s^−1^ to Equation (10). The results are depicted in Figure 11. Consequently, all parameters for the complete constitutive model of EPDM within the extended strain rate range were obtained and are listed in Table 3.

### 5.2. Model Validation

**Figure 12:**
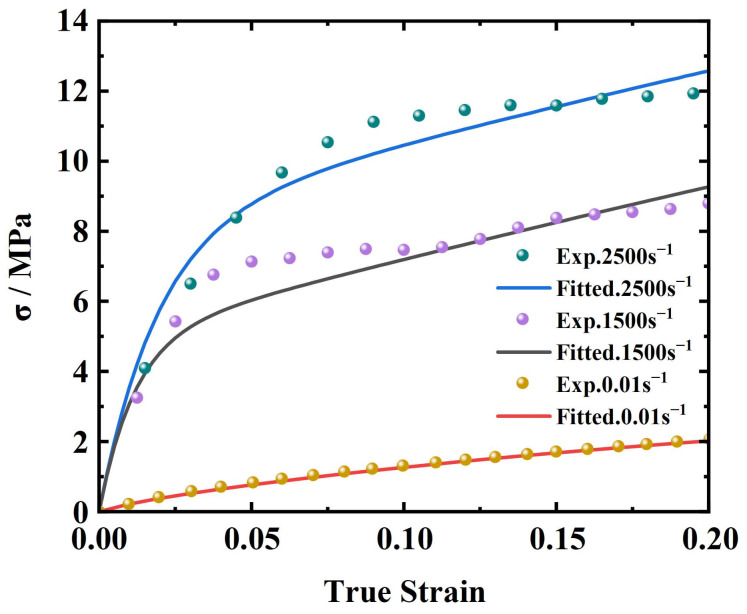
Comparison between model curves and test data.

As shown in Figure 12, the established constitutive model for EPDM coating was used to calculate the stress–strain results at strain rates of 0.01 s^−1^, 1500 s^−1^, and 2500 s^−1^. These results were then compared with the quasi-static and dynamic experimental data. It can be observed that some data points exhibit slight deviations from the fitted curve, potentially due to experimental errors, the nonlinear characteristics of the material, or limitations of the fitting model. However, overall, the predicted results align closely with the experimental data, and the fitted curve effectively reflects the trend of the experimental data. The model accurately captures the inflection points of the curves and describes the mechanical behavior within the 20% strain range. Therefore, the improved ZWT constitutive model developed in this study is capable of describing the tensile properties of EPDM coating over a wide range of strain rates.

## 6. Conclusions

In this study, the mechanical properties of aluminum-hydroxide-enhanced EPDM coating materials were obtained through quasi-static and dynamic experiments, leading to the development of an enhanced ZWT nonlinear viscoelastic constitutive model to describe the behavior of EPDM coating over a wide range of strain rates. The primary findings and contributions are summarized as follows:

The aluminum-hydroxide-enhanced EPDM coating of solid rocket motors was confirmed as a rate-dependent polymer composite material. Under both quasi-static and dynamic tensile conditions, stress increases with strain rate. Additionally, the stress at the inflection point on the dynamic strain–stress curve also increases with strain rate, indicating the material’s sensitivity to loading conditions.

To capture the hyperelastic and viscoelastic characteristics of aluminum-hydroxide-enhanced EPDM coating at larger strains, the standard ZWT model was modified. The improved ZWT nonlinear viscoelastic constitutive model effectively describes the tensile behavior of EPDM coatings within a strain range of 20% and a strain rate range of 10^−3^ to 2500 s^−1^. The model predictions closely matched the experimental data, accurately capturing the stress–strain response and the inflection points in the curves. The experimental data validated the model’s reliability.

The findings of this study contribute significantly to the understanding and simulation of aluminum-hydroxide-enhanced EPDM coatings. The proposed model offers a reliable method for predicting their mechanical behavior under varying strain rates, facilitating the development of accurate material simulation numerical methods. Future work will focus on exploring a broader range of strains and incorporating environmental factors such as temperature to further refine the model. This will enhance its applicability under complex conditions, ultimately improving the reliability and performance of EPDM coatings in aerospace applications.

## Figures and Tables

**Table 1:** Composition of EPDM specimens.

Component	EPDM	Al(OH)_3_	Flame Retardant	Processing Aid
Content (wt%)	56	30	10	4

**Table 2:** SHTB experiment device parameters.

Parameters	Striking Tube	Waveguide Bar	Incident Bar	Transmitted Bar
Material	TC4	TC4	TC4	PMMA
Length/m	0.6	2.5	1.7	2
Diameter/mm	27^outer^, 20^inner^	19	14	14
Modulus/MPa	113,500	113,500	113,500	4774
Wave speed/m/s	4966	4966	4966	2110

**Table 3:** All parameters of the proposed constitutive model.

*E*_1_	*θ*_1_	*α*_1_	*μ*_1_	*α*_2_	*μ*_2_	*α*_3_	*μ*_3_	*E*_2_	*θ*_2_
12.35	1.19	−5.57	6.31	−2.98	0.75	−8.34	−3.51	425.63	7.78 × 10^−6^

## Data Availability

The original contributions presented in this study are included in this article; further inquiries can be directed to the corresponding author.

## References

[1] Ahmed A.F., Hoa S.V. (2012). Thermal Insulation by Heat Resistant Polymers for Solid Rocket Motor Insulation. J. Compos. Mater..

[2] Amado J.C.Q., Ross P.G., Sanches N.B., Pinto J.R.A., Dutra J.C.N. (2020). Evaluation of Elastomeric Heat Shielding Materials as Insulators for Solid Propellant Rocket Motors: A Short Review. Open Chem..

[3] Wei B., Yu C., Bai Y., Liu L., He J. (2023). Preparation Optimization of CFRP and EPDM Composite by the Co-Curing Method. Materials.

[4] George K., Panda B.P., Mohanty S., Nayak S.K. (2018). Recent Developments in Elastomeric Heat Shielding Materials for Solid Rocket Motor Casing Application for Future Perspective. Polym. Adv. Technol..

[5] Sureshkumar M.S., Bhuvaneswari C.M., Kakade S.D., Gupta M. (2008). Studies on the Properties of EPDM–CSE Blend Containing HTPB for Case-bonded Solid Rocket Motor Insulation. Polym. Adv. Technol..

[6] Bhuvaneswari C.M., Sureshkumar M.S., Kakade S.D., Gupta M. (2006). Ethylene-Propylene Diene Rubber as a Futuristic Elastomer for Insulation of Solid Rocket Motors. Def. Sci. J..

[7] Liu Y., Li X., Zhu P., Xi K. (2022). Ablation Characteristics of Insulator under High-Temperature Gas Dual-Pulse Erosion. Def. Technol..

[8] Ye Q., Yu Y., Li W. (2020). Study on Cook-off Behavior of HTPE Propellant in Solid Rocket Motor. Appl. Therm. Eng..

[9] Jiang C., Jin Y., Gao J. (2021). Ablation and Thermal Insulation Properties of Silicone Rubber-Polyarylacetylene-Carbonwoven Laminates for Solid Rocket Motor. Plast. Rubber Compos..

[10] Wang J., Cao P., Wang X. (2023). Review of the Mechanical Properties and Numerical Simulation of Composite Solid Propellants. Materials.

[11] Marimuthu R., Nageswara Rao B. (2013). Development of Efficient Finite Elements for Structural Integrity Analysis of Solid Rocket Motor Propellant Grains. Int. J. Press. Vessels Pip..

[12] Wang R., Wang N., Gao J., Zhang Y., Zhang A., Wu Y. (2023). Friction-Induced Ignition Study of HTPB Propellant Based on a Coupled Chemo-Mechano-Thermodynamic Model under Ultrahigh Acceleration Overload Conditions. Case Stud. Therm. Eng..

[13] Pei S., Qiang H., Wang X., Li S. (2024). Mesoscopic Failure Behavior of HTPB Propellant Bonding Interface under Multi-Angle Pull-and-Shear Loading. Polym. Test..

[14] Zhang Y., Wang N., Ma W., Wang R., Bai L., Wu Y. (2024). Investigations of the Mechanical Response of Dummy HTPB Propellant Grain under Ultrahigh Acceleration Overload Conditions Using Onboard Flight-Test Measurements. Def. Technol..

[15] Liu H., Wang J., Fu X. (2024). Research on Crack Propagation of Nitrate Ester Plasticized Polyether Propellant: Experiments and Simulation. Materials.

[16] George K., Panda B.P., Biswal M., Mohanty S., Nayak S.K. (2020). Ethylene Propylene Diene Monomer Rubber-Based Heat Shielding Materials for Solid Rocket Motor: Impact of Kevlar Fiber Reinforcement on the Thermal and Mechanical Properties. Polym. Adv. Technol..

[17] Li J., Liu K., Guo M., Liu Y., Wang J., Lv X. (2018). Ablation and Erosion Characteristics of EPDM Composites under SRM Operating Conditions. Compos. Part Appl. Sci. Manuf..

[18] Sapozhnikov I., Leitner A., Natan B., Mograbi E. (2021). Investigation of the Ablative Properties of an EPDM\Kevlar Insulator in a Solid Rocket Motor. Propellants Explos. Pyrotech..

[19] Jia X., Li G., Sui G., Li P., Yu Y., Liu H., Yang X. (2008). Effects of Pretreated Polysulfonamide Pulp on the Ablation Behavior of EPDM Composites. Mater. Chem. Phys..

[20] Jia X., Li G., Yu Y., Sui G., Liu H., Li Y., Li P., Yang X. (2009). Ablation and Thermal Properties of Ethylene–Propylene–Diene Elastomer Composites Reinforced with Polysulfonamide Short Fibers. J. Appl. Polym. Sci..

[21] Gao G., Zhang Z., Li X., Meng Q., Zheng Y. (2010). An Excellent Ablative Composite Based on PBO Reinforced EPDM. Polym. Bull..

[22] Vishvanathperumal S., Roy J.V., Anand G., Ramu K.N., Praveenkumar S. (2024). An Investigation on the Effect of the Surface Modifications and HNTs Loading on the Cure Behaviours, Abrasion Resistance, Mechanical and Morphological Properties of NR/EPDM Nanocomposites. Silicon.

[23] Arshad N., Qasim G., Beagan A.M. (2022). Investigations on the Morphological, Mechanical, Ablative, Physical, Thermal, and Electrical Properties of EPDM-Based Composites for the Exploration of Enhanced Thermal Insulation Potential. Polymers.

[24] Natali M., Rallini M., Kenny J., Torre L. (2016). Effect of Wollastonite on the Ablation Resistance of EPDM Based Elastomeric Heat Shielding Materials for Solid Rocket Motors. Polym. Degrad. Stab..

[25] Zhang G., Wang F., Huang Z., Dai J., Shi M. (2016). Improved Ablation Resistance of Silicone Rubber Composites by Introducing Montmorillonite and Silicon Carbide Whisker. Materials.

[26] Fan X., Xu J., Chen X., Wang S., Hu S. (2019). Investigation on Failure Properties and Constitutive Modeling of EPDM Used for Pulse Separation Device. Mech. Mater..

[27] Jiang J., Xu J., Zhang Z., Chen X. (2016). Rate-Dependent Compressive Behavior of EPDM Insulation: Experimental and Constitutive Analysis. Mech. Mater..

[28] Wang S., Xu J., Li H., Liu J., Zhou C. (2022). The Effect of Thermal Aging on the Mechanical Properties of Ethylene Propylene Diene Monomer Charge Coating. Mech. Time-Depend. Mater..

[29] Song B., Chen W. (2003). One-Dimensional Dynamic Compressive Behavior of EPDM Rubber. J. Eng. Mater. Technol..

[30] Cheng M., Chen W. (2003). Experimental Investigation of the Stress–Stretch Behavior of EPDM Rubber with Loading Rate Effects. Int. J. Solids Struct..

[31] Enew A.M., Elfattah M.A., Fouda S.R., Hawash S.A. (2021). Effect of Aramid and Carbon Fibers with Nano Carbon Particles on the Mechanical Properties of EPDM Rubber Thermal Insulators for Solid Rocket Motors Application. Polym. Test..

[32] Li C., Guo J., Xu P., Hu W., Lv J., Shi B., Zhang Z., Li R. (2023). Facile Preparation of Superior Compressibility and Hydrophobic Reduced Graphene Oxide@cellulose Nanocrystals/EPDM Composites for Highly Efficient Oil/Organic Solvent Adsorption and Enhanced Electromagnetic Interference Shielding. Sep. Purif. Technol..

[33] Xu P., Lv J., Guo J., Hou D., Zhang L., Sun Y., Li R., Li C. (2023). Preparation of EPDM/Silicon Nanofibers-Graphene Nanocomposites with Enhanced Interfacial Structure: Highly Reinforcing and Stabilizing Effect. Diam. Relat. Mater..

[34] Nagata K., Takahashi Y., Shibusawa S., Nakamura Y. (2002). Interfacial Structure in Vulcanized EPDM Filled with Mercaptosilane-Treated Al(OH)_3_ and Its Influence on the Mechanical Properties. J. Adhes. Sci. Technol..

[35] Su Z., Jiang P., Li Q., Wei P., Zhang Y. (2005). Toughening of Polypropylene Highly Filled with Aluminum Hydroxide. Polym. Polym. Compos..

[36] Yen Y.-Y., Wang H.-T., Guo W.-J. (2013). Synergistic Effect of Aluminum Hydroxide and Nanoclay on Flame Retardancy and Mechanical Properties of EPDM Composites. J. Appl. Polym. Sci..

[37] Li M., Li Z., He H., Chen L., Miao Y. (2022). Mechanical Behaviors and Constitutive Relations under Wide Strain Rate Range for CMDB Propellant. Polym. Test..

[38] Miao Y.G. (2018). On Loading Ceramic-like Materials Using Split Hopkinson Pressure Bar. Acta Mech..

[39] Miao Y., Yin J., Du W., Chen L. (2024). Mechanical Behavior of Nanorubber Reinforced Epoxy over a Wide Strain Rate Loading. Nano Mater. Sci..

[40] Miao Y., Wang Y., Du W., He H., Deng Q., Dou Q. (2022). Theoretical Instructions for Experimenting Controllable Hopkinson Pressure Bar. Polym. Test..

[41] Xu Z.-J., Li Y.-L., Huang F.-L. (2012). Application of Split Hopkinson Tension Bar Technique to the Study of Dynamic Fracture Properties of Materials. Acta Mech. Sin..

[42] Chen W., Lu F., Frew D.J., Forrestal M.J. (2002). Dynamic Compression Testing of Soft Materials. J. Appl. Mech..

[43] Zhu J., Hu S., Wang L. (2009). An Analysis of Stress Uniformity for Concrete-like Specimens during SHPB Tests. Int. J. Impact Eng..

[44] Chen C., Li Y., Chang M., Guo K., Han Y.F., He L., Xu M., Tang E. (2022). Zhu-Wang-Tang Constitutive Model of Reinforced Al/PTFE Materials at Medium Strain Rate. Polym. Compos..

[45] Gao G., Tang E., Yang G., Han Y., Chen C., Chang M., Guo K., He L. (2024). Parameter Determination and Verification of ZWT Viscoelastic Dynamic Constitutive Model of Al/Ep/W Material Considering Strain Rate Effect. Int. J. Impact Eng..

[46] Wang R., Tang E., Yang G., Sun Q., Han Y., Chen C. (2021). Construction of PZT-5H Mechano-Electric Model Based on Strain Rate Dependence and Its Numerical Simulation in Overload Igniter Application. Mech. Mater..

[47] Wang L., Labibes K., Azari Z., Pluvinage G. (1994). Generalization of Split Hopkinson Bar Technique to Use Viscoelastic Bars. Int. J. Impact Eng..

[48] Kossa A., Valentine M.T., McMeeking R.M. (2023). Analysis of the Compressible, Isotropic, Neo-Hookean Hyperelastic Model. Meccanica.

[49] Ogden R.W. (1972). Large Deformation Isotropic Elasticity—On the Correlation of Theory and Experiment for Incompressible Rubberlike Solids. Proc. R. Soc. Math. Phys. Eng. Sci..

[50] Yeoh O.H. (1990). Characterization of Elastic Properties of Carbon-Black-Filled Rubber Vulcanizates. Rubber Chem. Technol..

[51] Zhao G., Ma Y., Li Y., Luo J., Du C. (2017). Development of a Modified Mooney-Rivlin Constitutive Model for Rubber to Investigate the Effects of Aging and Marine Corrosion on Seismic Isolated Bearings. Earthq. Eng. Eng. Vib..

[52] Gomez-Jimenez S., Saucedo-Anaya T., Guerrero-Mendez C., Robles-Guerrero A., Silva-Acosta L., Navarro-Solis D., Lopez-Betancur D., Contreras Rodríguez A.R. (2024). Mooney–Rivlin Parameter Determination Model as a Function of Temperature in Vulcanized Rubber Based on Molecular Dynamics Simulations. Materials.

[53] Anssari-Benam A., Destrade M., Saccomandi G. (2022). Modelling Brain Tissue Elasticity with the Ogden Model and an Alternative Family of Constitutive Models. Philos. Trans. R. Soc. Math. Phys. Eng. Sci..

[54] Kim B., Lee S.B., Lee J., Cho S., Park H., Yeom S., Park S.H. (2012). A Comparison among Neo-Hookean Model, Mooney-Rivlin Model, and Ogden Model for Chloroprene Rubber. Int. J. Precis. Eng. Manuf..

[55] Gad A.G. (2022). Particle Swarm Optimization Algorithm and Its Applications: A Systematic Review. Arch. Comput. Methods Eng..

[56] Wang D., Tan D., Liu L. (2018). Particle Swarm Optimization Algorithm: An Overview. Soft Comput..

